# Supplementary material for: DMRforPairs: identifying Differentially Methylated Regions between unique samples using array based methylation profiles
Source: BMC Bioinformatics. 2014 May 15;15:141. doi: 10.1186/1471-2105-15-141 (PMC4046028; doi:10.1186/1471-2105-15-141)
Supplement: Additional file 2 — DMRforPairs output for the comparison of A431-MCF7 and NA17018-NA17105. Please start from the HTML files in each folder. Available via the BMC Bioinformatics website. [file 1471-2105-15-141-S2.zip › 1394847754114233_MOESM2_ESM/A431_MCF7/figures/16657.pdf]

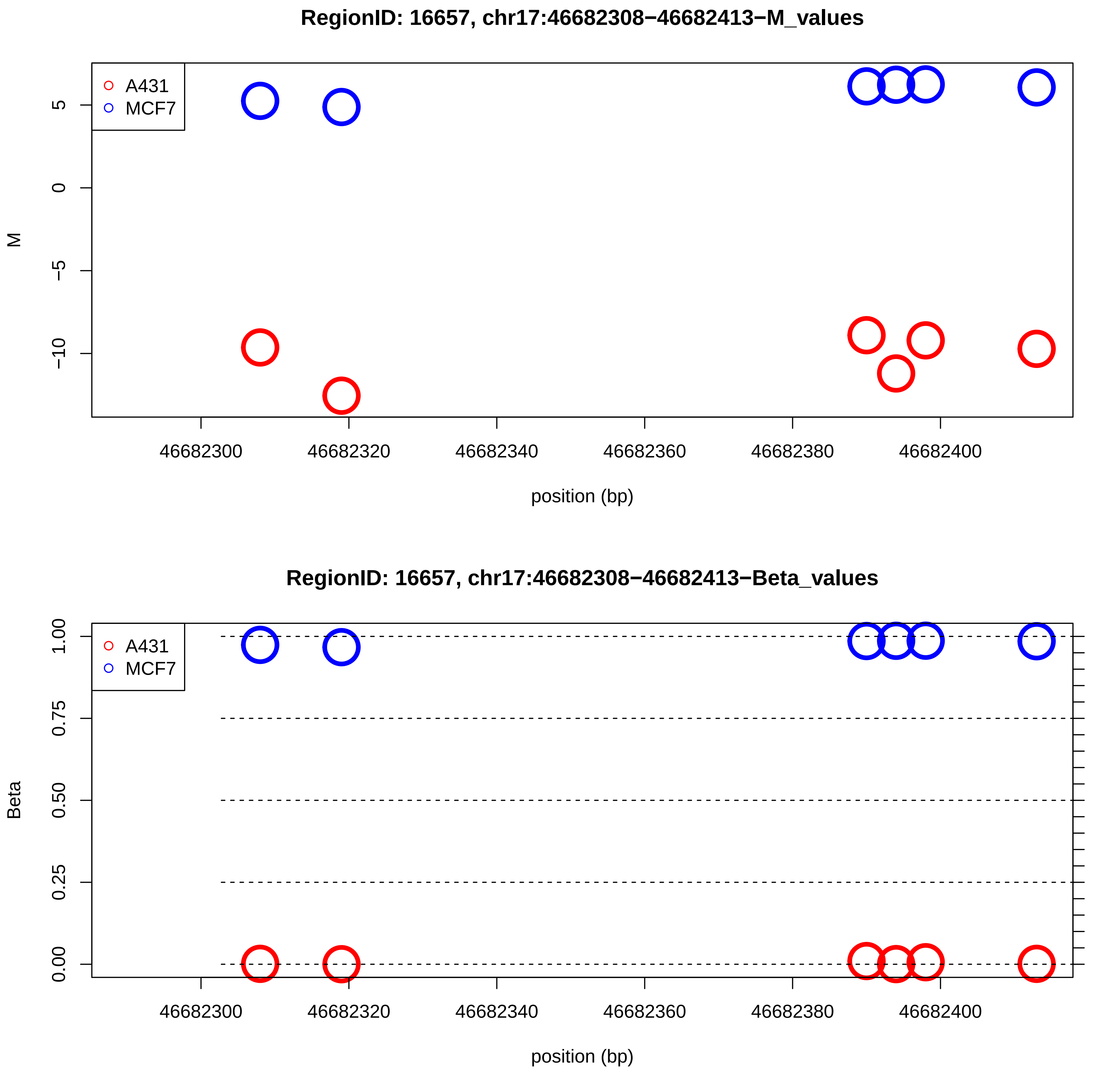
RegionID: 16657, chr17:46682308−46682413−M_values
A431
MCF7
5
0
−5
−10
M
46682300
46682320
46682340
46682360
46682380
46682400
position (bp)
RegionID: 16657, chr17:46682308−46682413−Beta_values
A431
MCF7
1.00
0.75
0.50
0.25
0.00
Beta
46682300
46682320
46682340
46682360
46682380
46682400
position (bp)

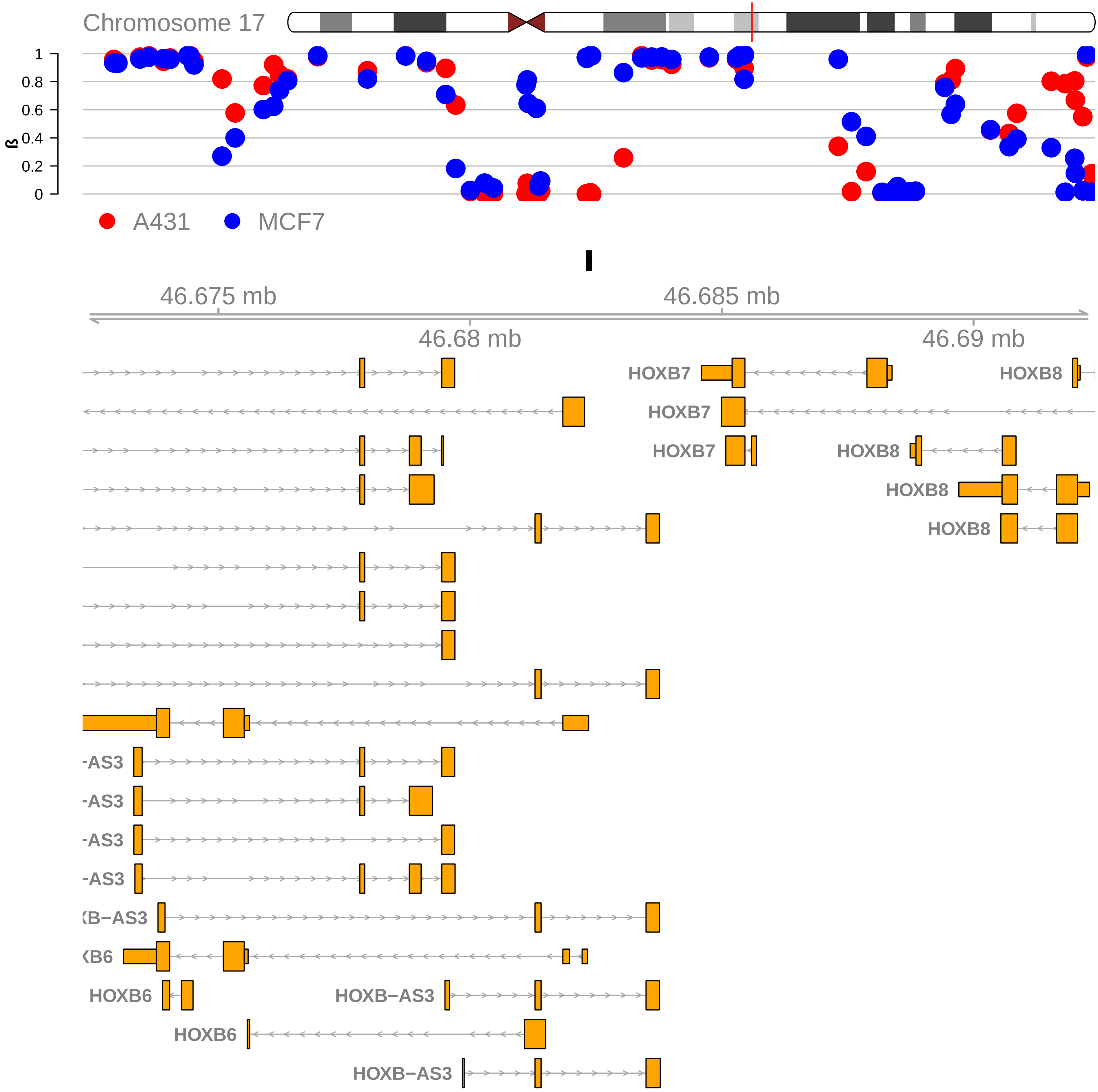

Chromosome 17
1
0.8
0.6
0.4
0.2
0
ß
A431
MCF7
46.675 mb
46.685 mb
46.68 mb
46.69 mb
HOXB7
HOXB8
HOXB7
HOXB7
HOXB8
HOXB8
HOXB8
-AS3
-AS3
-AS3
-AS3
(B−AS3
(B6
HOXB6
HOXB−AS3
HOXB6
HOXB−AS3
